# Use of the Online Portal “Embryotox” in Routine Health Care: Mixed Methods Study

**DOI:** 10.2196/81286

**Published:** 2026-06-25

**Authors:** Marlies Onken, Evelin Beck, Martina Breuning, Christine Holmberg, Franziska Metke, Anne Müller, Christof Schaefer, Katarina Dathe

**Affiliations:** 1 Charité – University Medical Center Berlin, corporate member of Freie Universität Berlin and Humboldt-Universität zu Berlin, Institute of Clinical Pharmacology and Toxicology, Embryotox Center of Clinical Teratology and Drug Safety in Pregnancy, Augustenburger Platz 1, Berlin, 13353, Germany, 49 030 450 525 702, 49 030 450 7525729; 2 Institute of Social Medicine and Epidemiology, Brandenburg Medical School Theodor Fontane, Brandenburg an der Havel, Germany

**Keywords:** pregnancy, breastfeeding, drug safety, risk assessment, consumer health information, online health information, shared decision-making

## Abstract

**Background:**

Since 2008, the open-access internet portal “Embryotox” has provided evidence-based information on drug safety during pregnancy and breastfeeding. The German website, designed for health care professionals, comprises fact sheets about 400 drugs and is maintained by the Embryotox Center of Clinical Teratology and Drug Safety in Pregnancy at the Charité – Universitätsmedizin Berlin. In 2024, it was accessed more than 5 million times.

**Objective:**

This study aimed to evaluate who is using the website, how it is being used, and to what extent it meets users’ needs.

**Methods:**

Data were collected between 2022 and 2023 through a sequential mixed methods design incorporating explanatory and exploratory elements. Online questionnaire 1 contained questions on user characteristics, clinical specifics of use, comprehensibility, and changes in risk assessment or drug therapy following fact sheet use. Qualitative data were generated through semistructured phone interviews, with interviewees sampled from the main user groups identified in questionnaire 1 (patients, physicians, pharmacists, and midwives). Kuckartz’s content analysis was used to analyze data on users’ trust and the functions of website use for different stakeholder groups. Online questionnaire 2 collected data from the main user groups on their decision-making based on Embryotox fact sheets. This questionnaire was developed based on the findings from semistructured interviews on the functions of website use. Answers in both questionnaires included multiple-choice options or ratings on a Likert scale from 0 (not at all) to 10 (fully); data were analyzed using descriptive statistics.

**Results:**

Questionnaire 1 was completed by 14,562 users, including 10,860 patients, 1676 physicians, 550 pharmacists, and 364 midwives. Of the physicians, 27.2% (456/1676) were obstetricians and gynecologists, and 22.7% (381/1676) were general practitioners. For physicians, the mean of comprehensibility ratings was 9.39 (pharmacists 9.25; midwives 9.14), and for patients 8.80. Following fact sheet use, 66.6% (9694/14,562) of all users changed their risk perception, and 22.3% (2252/10,083) of users in a specific treatment setting considered changing drug treatment. Qualitative content analysis revealed that users highly trusted the information in the fact sheets for a number of reasons, including recommendations from other users, the scientific basis of the website, and the authority of the Embryotox Center as a university-based specialist department for drug safety in pregnancy and lactation. Functions of fact sheet use differed depending on the stakeholder group. A total of 793 users, mostly physicians and patients, fully completed questionnaire 2, specifying typical clinical situations and benefits of fact sheet use for shared decision-making. Benefits included facilitating the decision-making process and increasing confidence in it.

**Conclusions:**

The information provided in the Embryotox fact sheets was generally perceived as both comprehensible and trustworthy, supporting and facilitating the decision-making process. Embryotox is used successfully by health care providers and patients in routine health care settings, helping to improve drug safety for pregnant and breastfeeding women.

## Introduction

Medical publications have multiplied in recent decades and continue to do so. This has created a constant challenge to ensure quality standards for routine health care in interdisciplinary areas such as drug safety in pregnant and breastfeeding women. In a French drug utilization study, about 90% (32,408/36,065) of pregnant women used prescription drugs [1] to treat preexisting or newly occurring maternal conditions. However, in this vulnerable stage, the use of medicines is fraught with considerable uncertainty. Inadequate knowledge often leads to an overestimation of drug risks, both by health care professionals (HCPs) and patients. This can have far-reaching consequences such as nonadherence leading to inadequately treated maternal conditions [2], premature discontinuation of breastfeeding, or even termination of an otherwise desired pregnancy. Conversely, underestimating the risks of medicines can lead to a failure to switch to a more appropriate treatment or to use effective contraception. Information on reproductive health is regularly searched for on the internet [3-7], and the dangers of misinformation from online health information are widely discussed [8,9]. Online information on perinatal drug safety is also often contradictory or misleading, especially when different sources are compared [10-12]. Comprehensive and reliable evidence-based information is therefore urgently needed [13-15]. Beginning in the 1980s, multidisciplinary, often publicly funded institutions have been established to provide information on drug safety in pregnancy and breastfeeding in many countries (see, eg, [16] or [17]). Initially, information was mainly offered via telephone counseling, but with the advent of mass internet use, some centers have also started to provide online information. Table S1 in Multimedia Appendix 1 provides examples of evidence-based online resources on drug safety in pregnancy and breastfeeding in various languages.

In Germany, the Berlin Embryotox Center was founded in 1988 [18], offering telephone counseling on the risk and safety of drugs in pregnancy and breastfeeding for HCPs and patients. The number of counseling requests has now settled at around 12,000 per year, a quarter of them by HCP. Also, the Embryotox Center collects and scientifically analyzes data on exposed pregnancies (“Embryotox cohort”), partly in collaboration with the European Network of Teratology Information Services [19,20], an umbrella organization established in 1990. A website with online information was launched in 2008 [21], cofunded by the German Federal Ministry of Health. This online resource was intended for use by HCPs, but has been publicly accessible from the outset. It now contains fact sheets on approximately 400 drugs, with each fact sheet providing the following information: (1) brief introduction to the drug, (2) a color code labeling (green, gray, and red) to summarize risk assessment, (3) description of scientific evidence for both first trimester and second and third trimester use, (4) clinical recommendations and consequences, including more adequate treatment alternatives if appropriate, and (5) scientific evidence and clinical recommendations for use during breastfeeding. Embryotox also offers fact sheets on the course and treatment of selected diseases during pregnancy and breastfeeding. Furthermore, the website provides information on the Embryotox Center*,* such as contact details for counseling, information on staff members, ongoing and past studies and scientific projects, including a list of scientific publications.

The rapidly increasing number of website users—growing from fewer than 200,000 visits in 2009 to more than 5.1 million in 2024—suggested early on that Embryotox was widely used not only by HCPs but also by patients. In order to tailor the information to the needs of its users in everyday medical practice, there was an urgent need to evaluate the use and perception of the website and to analyze how and by whom the fact sheets are used in routine health care. This mixed methods study was conducted to answer these questions.

## Methods

### Overview

This mixed methods study used a sequential design (quan-qual-quan), incorporating both explanatory and exploratory elements [22,23] to gain insight into the use of Embryotox in routine health care. Some of the quantitative findings from the first online questionnaire contributed to the development of the qualitative interview guides, while others, such as the demographic and occupational information, did not require in-depth qualitative exploration. The qualitative interviews, in turn, formed the basis for developing the second quantitative online questionnaire, which focused on a subtopic of Embryotox use, namely the use for clinical decision-making. Quantitative data from both online questionnaires are reported in accordance with the CHERRIES (Checklist for Reporting Results of Internet E-Surveys) statement [24]; the completed checklist is included in Checklist 1. Reporting of qualitative data follows the COREQ (Consolidated Criteria for Reporting Qualitative Research) checklist [25]. The completed COREQ checklist can be found in Checklist 2.

### Collection of Quantitative Data

#### Overview

The research team developed 2 online questionnaires and tested them among colleagues from both academic and nonacademic backgrounds, as well as among individuals from their social circles. Technical details were worked out in collaboration with the digital agency that technically manages the website and also programmed both questionnaires. Visits to Embryotox drug fact sheets were counted via an anonymous session-tracking procedure by a commercial provider. To identify potential multiple entries, datasets of questionnaires were analyzed regarding entry times and similarity of entries. IP addresses or cookies were not used for this purpose. In accordance with the General Data Protection Regulation in Germany, questionnaires were designed in such a way that no conclusions could be drawn about individuals. Banner ads leading to the questionnaires were based directly on the drug fact sheets. The banner leading to the first questionnaire (2022) invited users to participate with the words “We ask for your feedback.” On the banner leading to the second questionnaire (2023), users were asked, “How do you use embryotox.de? Questionnaire about your experiences.” Participation was voluntary and not a prerequisite for using the drug fact sheets. According to their answers, participants were automatically guided through the questionnaire. Clicking back was not possible. Progress within the questionnaire was visible via a bar. Only one question at a time displayed on the screen, and participants could move on to the next question only after answering the previous question. Only the question about the educational qualifications of lay users was optional and could be skipped. The selection of mutually exclusive answer options and the selection of more than one answer (if not explicitly requested) were blocked. The answers were only saved if the users completed the questionnaire to the end and then selected the button “Send data.”

#### Online Questionnaire 1: Use and Users of Embryotox in Routine Health Care

Online questionnaire 1 was placed on all drug fact sheets for 7 months (May-December 2022). Over the same period, the total number of visits to each fact sheet was counted. Users were asked to complete the questionnaire only once. Questionnaire 1 included questions on user characteristics, in particular demographic information (age and gender) and, for HCP, occupational information such as profession (for physicians also specialization) and working area (outpatient care, hospital, pharmacy, and other). Patients and other non-HCPs were asked about their level of education and how they got to know about Embryotox. HCPs were asked whether they were looking for information for a specific patient and whether they had found the essential information they needed to make a well-founded treatment decision. All users were asked to specify the frequency and duration of Embryotox use and the clinical focus of their search. Furthermore, all users were asked to evaluate the comprehensibility of the fact sheet they had accessed and whether its information resulted in changes in risk perception or preferred medication. Possible answers comprised either multiple-choice options or ratings on a Likert scale ranging from 0 (not at all) to 10 (fully). For a detailed flow chart of questionnaire 1, refer to Multimedia Appendix 2.

In addition, weekday, daytime, and the accessed fact sheet were recorded.

#### Online Questionnaire 2: Fact Sheet Use for Decision-Making

Questionnaire 2 was placed for 7 months (February-September 2023) on fact sheets for prescription-only drugs that typically require shared decision-making (refer to Table S3 in Multimedia Appendix 1). The questionnaire was aimed at the main user groups identified in questionnaire 1, primarily at physicians and patients as the main decision-makers. For the detailed flowchart, refer to Multimedia Appendix 3. The questionnaire was developed based on the findings from semistructured interviews on the functions of website use. For all user groups, answers comprised multiple-choice options or ratings on a Likert scale ranging from 0 to 10 or from “strongly agree” via “moderately agree” and “moderately disagree” to “strongly disagree.” If appropriate, “question not applicable” was also an answer option.

Questions and answer options were tailored to the respective user groups. Physicians answered up to 15 questions focusing on (1) their medical specialization, (2) typical use of fact sheets (eg, with or without patient, during or outside of consultation; maximum 3 out of 7 possible answers), (3) fact sheet use in the process of risk-benefit assessment and decision-making (eg, risk assessment and decision with or without patient, and satisfaction with fact sheet use), (4) further effects and functions of fact sheet use (eg, perceived effects on patient adherence and confidence in decision-making), and (5) potentially useful supplementary materials to the information provided on the website (maximum 2 out of 7 possible answers). Patients answered up to 16 questions about (1) fact sheet use for risk-benefit assessment and decision-making, (2) potential benefit or harm from the use of fact sheets, (3) effects and functions of fact sheet use, (4) potentially missing information, and (5) involvement of other persons in the decision-making process (multiple answers possible). Pharmacists and midwives answered up to 6 questions about the clinical situation, their typical fact sheet use (maximum 3 out of 5 possible answers) and functions of fact sheet use.

### Analysis of Quantitative Data

Descriptive statistics were used to analyze the quantitative data from both online questionnaires. The statistical analyses were conducted using R (R Core Team; version 4.4.0) [26]. To obtain additional information on fact sheet use, the weekday and time of day that each questionnaire 1 was completed were also analyzed. Additionally, the total number of visits to each fact sheet while questionnaire 1 was available online was used to calculate the proportion of completed questionnaire 1 in relation to the total number of fact sheet uses.

### Collection of Qualitative Data

Qualitative data on the use of Embryotox were obtained through semistructured phone interviews with individuals from the main user groups identified in questionnaire 1, that is, patients, physicians, pharmacists, and midwives. The interview guides were developed based on the aims of the study and the results from questionnaire 1. They aimed to provide insight into how Embryotox is used in various clinical settings and by different stakeholders within routine health care. Interview guides were piloted with each user group, after which the research team discussed and agreed upon any necessary adjustments by consensus. The guides focused on the functions of fact sheet use, as well as on the usability of Embryotox and possible needs for optimization. All interviewees were asked whether they trusted the information on Embryotox, and if they answered in the affirmative, to explain their reasons. Selected topics from questionnaire 1, such as comprehensibility, were also included for further exploration. For an overview, refer to Table S2 in Multimedia Appendix 1. Interviewees were recruited by purposive and snowball sampling. The aim of the sampling was to find interview participants who frequently use Embryotox and to achieve broad representation of the users’ experiences. All interviews were carried out by the same interviewer (AM, female). Data saturation was assessed with each new interview within a user group, as this has been suggested as a method of determining the sample size for qualitative interviews [27]. Data saturation was achieved for a user group when few or no new topics had emerged in the last 3 interviews. The interviews lasted about 30 minutes on average.

### Analysis of Qualitative Data

Kuckartz’s content analysis with a deductive-inductive approach was used to analyze qualitative data [28]. The f4 software (dr. dresing & pehl GmbH) [29] was used for transcription, coding, and analysis of the interviews. First deductive categories were derived from the interview guide and the research question and discussed in the research team. In addition to the deductive categories, further categories and subcategories were determined inductively from the material. The coding tree and themes were developed based on the first 3 interviews; they were double-checked and discussed by the research team to ensure intersubjective comprehensibility. Any disagreements were resolved through discussion within the research team [30]. Subsequent interviews were coded and analyzed using this coding structure in the f4analyse software (dr. dresing & pehl GmbH) [29]. For the following evaluation, we analyzed user statements on (1) trust in fact sheet information and (2) functions of fact sheet use, including the purpose and clinical utility of typical use.

### Ethical Considerations

Ethical approval for the collection and analysis of qualitative and quantitative data was obtained from the Ethical Committee of the Charité - Universitätsmedizin Berlin (EA1/334/21). For participating in the online survey, no formal written consent was required. Participants in the qualitative phone interviews signed a consent form following verbal and written information. Participants were not compensated for completing the online questionnaires or taking part in the interviews. The online questionnaires did not request personal data that would allow for identification of the participants. Identifying personal data of the interviewees was removed during the transcription process.

## Results

### Overview

A total of 14,562 completed questionnaire 1 (use of Embryotox in routine health care) and 793 completed questionnaire 2 (fact sheet use for decision-making) were analyzed using descriptive statistics. A total of 41 semistructured telephone interviews formed the dataset for qualitative content analysis (reasons to trust and to use Embryotox).

### Use of Embryotox in Routine Health Care (Questionnaire 1)

#### Overview

Of the 14,562 completed questionnaire 1, 10,860 were filled by patients and 1676 by physicians, the latter mostly obstetricians and gynecologists (27.2%, 456/1676) or general practitioners (22.7%, 381/1676). Details of participating user groups are presented in Table 1.

**Figure 1.**
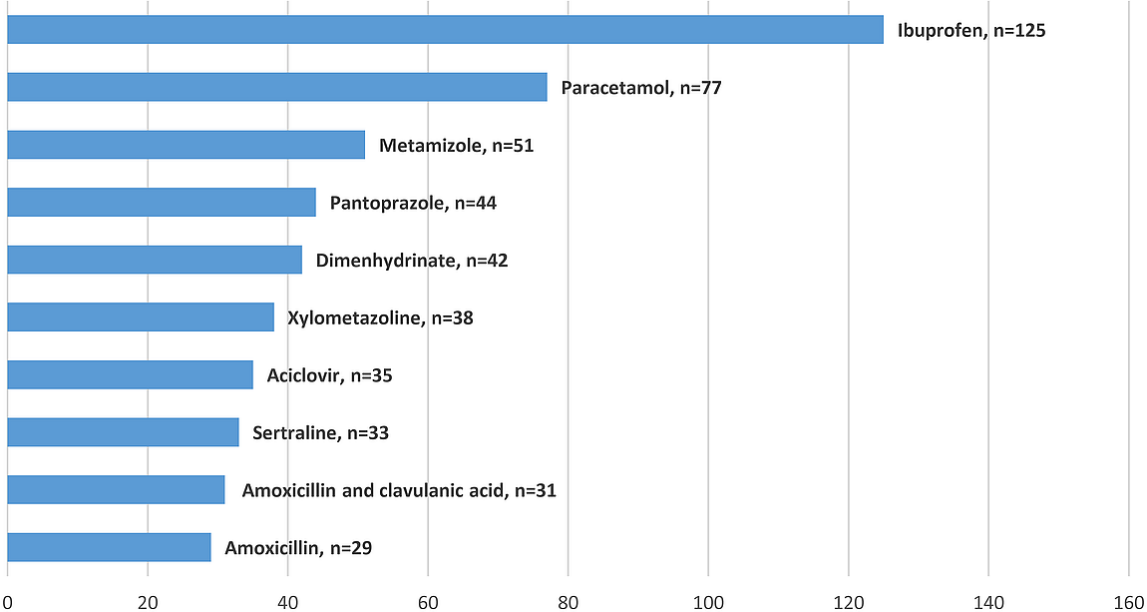
Top 10 drug fact sheets most frequently accessed by physicians (n=1676).

Overall, a median of 0.58% (IQR 0.42%‐0.77%) of visits to a drug fact sheet resulted in the completion of a questionnaire. The top 10 most frequently reviewed fact sheets are presented in Figures1  2 for physicians and patients, respectively.

**Table 1.** User groups contributing to questionnaire 1.

Completed questionnaires by user group (N=14,562)	Value, n (%)
Patients	10,860 (74.6)
Physicians	1676 (11.5)
Medical specialties:	
Obstetrics and gynecology	456 (27.2)
General practitioner	381 (22.7)
Internal medicine	214 (12.8)
Psychiatry, psychotherapy, or psychosomatics	129 (7.7)
Neurology	43 (2.6)
Pediatrics	93 (5.5)
Human genetics	3 (0.2)
Dentistry	17 (1.0)
Other medical specialties	340 (20.3)
Pharmacists	550 (3.8)
Midwives	364 (2.5)
Other health care professionals (eg, nurse, medical, or dental assistant)	324 (2.2)
Partner, relative, or friend of a patient	379 (2.6)
Other context	292 (2)
Student of medicine or other health sciences	117 (0.8)

**Figure 2.**
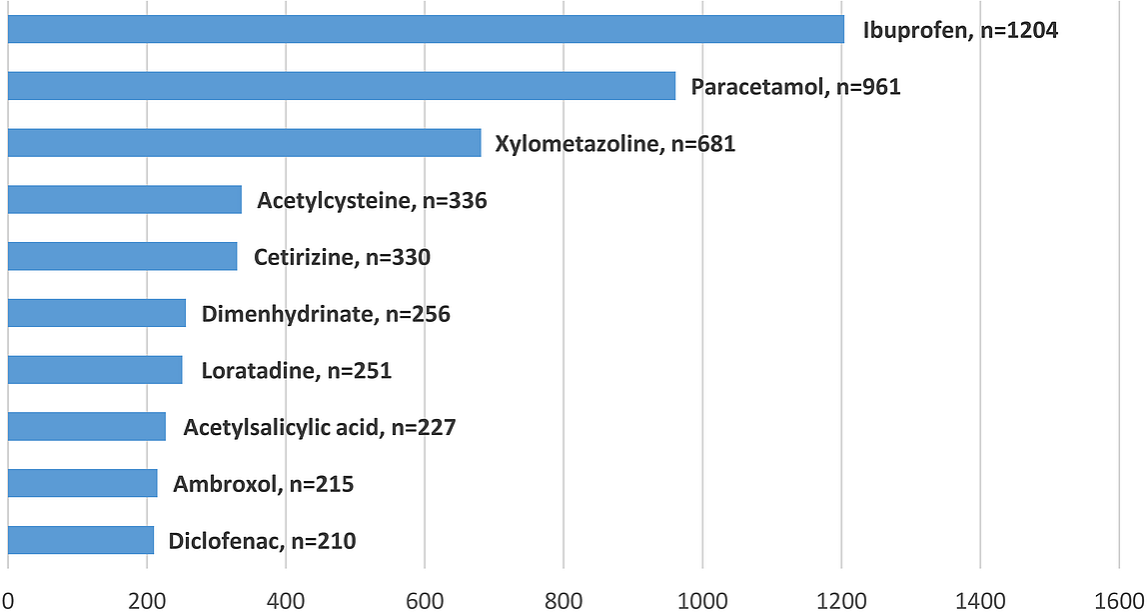
Top 10 drug fact sheets most frequently accessed by patients (n=10,860).

#### User Characteristics

**Figure 3.**
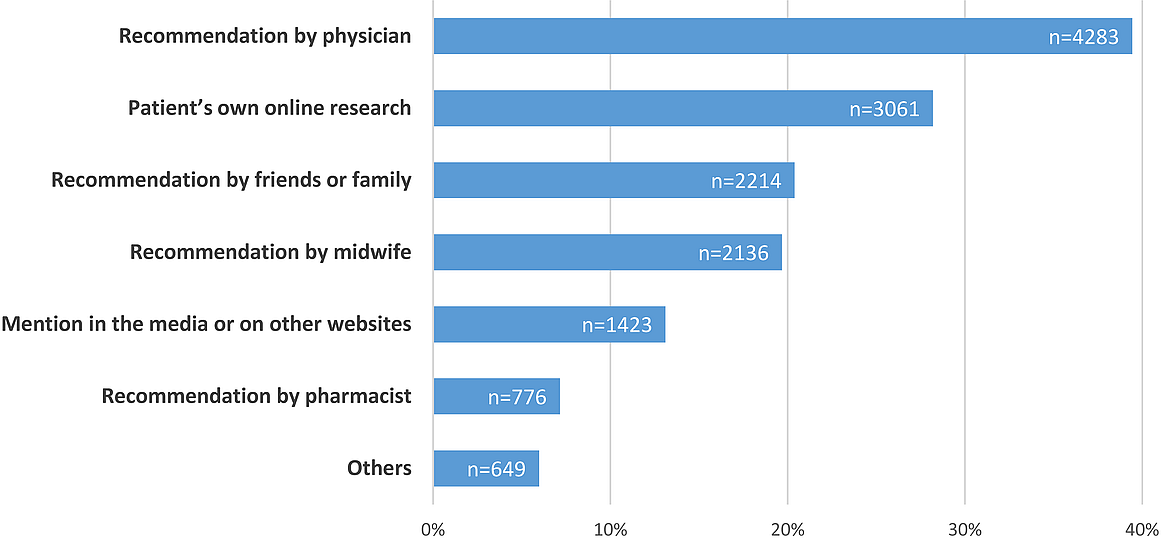
How patients (n=10,860) became aware of Embryotox.

For user characteristics of HCP, refer to Table 2. The majority of participating patients were aged between 31 and 40 years (69.3%, 7525/10,860), most of them had a high level of education (university degree 58.2%, 6319/10,860; qualification for higher education 20.7%, 2247/10,860; school-leaving certificate 10th or 11th grade 17.4%, 1887/10,860; school-leaving certificate 9th grade 2.3%, 247/10,860; no educational qualification or not specified: 1.5%, 160/10,860). Figure 3 shows how patients became aware of Embryotox.

**Table 2.** User characteristics of health care professionals (questionnaire 1).

Participants' characteristics	Physicians (n=1676)	Pharmacists (n=550)	Midwives (n=364)	Other HCP^a^ (n=324)
Age (years), n (%)				
≤30	140 (8.4)	153 (27.8)	62 (17)	122 (37.7)
31‐40	673 (40.2)	229 (41.6)	101 (27.7)	134 (41.4)
41‐50	415 (24.8)	75 (13.6)	118 (32.4)	45 (13.9)
51‐60	298 (17.8)	63 (11.5)	72 (19.8)	17 (5.2)
>60	150 (8.9)	30 (5.5)	11 (3)	6 (1.9)
Gender, n (%)				
Female	1093 (65.2)	468 (85.1)	361 (99.2)	284 (87.7)
Male	575 (34.3)	82 (14.9)	1 (0.3)	38 (11.7)
Diverse	8 (0.5)	0 (0)	2 (0.5)	2 (0.6)
Working area, n (%)				
Hospital	725 (43.3)	30 (5.5)	116 (31.9)	163 (50.3)
Outpatient care	893 (53.3)	0 (0)	173 (47.5)	87 (26.9)
Pharmacy	3 (0.2)	480 (87.3)	0 (0)	9 (2.8)
Other	55 (3.3)	40 (7.3)	75 (20.6)	65 (20.1)

a HCP: health care professional.

#### Usage of Fact Sheets

Drug fact sheets were accessed to a considerable extent outside standard working hours, that is, between 8 PM and 7:59 AM (physicians: 30.4%, 509/1676; patients: 42.9%, 4655/10,860; pharmacists: 28.4%, 156/550; midwives: 29.4%, 107/364). Most of the participating HCP stated that they had used the same fact sheet more than once (physicians: 73.3%, 1229/1676; pharmacists: 69.1%, 380/550; midwives: 77.5%, 282/364), as did the majority of patients (71%, 7708/10,860). At the time of fact sheet access, most of the participating patients were pregnant (46.8%, 5085/10,860) or were breastfeeding (42.8%, 4650/10,860). A total of 6% (649/10,860) of patients and 9.4% (131/1388) of physicians seeking information for a specific patient accessed Embryotox when pregnancy was still being planned. Among psychiatrists, the proportion was even higher (25.2%, 27/107). Details about fact sheet use by different user groups are presented in Table S4 in Multimedia Appendix 1.

#### Comprehensibility and Relevance

For all user groups combined (N=14,562), the mean of comprehensibility ratings was 8.88 (Likert scale from 0 to 10). For all HCPs, the mean of comprehensibility ratings was higher than 9 (physicians: 9.39, pharmacists: 9.25, midwives: 9.14, and other HCP: 9.01). For patients, the mean was 8.80, and for other lay persons (partners, relatives, and friends), it was 8.83. Patients’ assessment of comprehensibility depended on their level of education. The mean comprehensibility ratings was 7.29 for patients without a school-leaving certificate, while it was 8.92 for patients with an academic degree.

HCPs were asked to indicate if they had found the information required to make a well-founded treatment decision. This was answered in the affirmative (“Yes, definitely” and “Somewhat”) by 94.1% (1577/1676) of physicians, 92.7% (510/550) of pharmacists, and 92.6% (337/364) of midwives, with the majority of users fully agreeing (“Yes, definitely”: physicians 72.1%, 1208/1676; pharmacists 65.3%, 359/550; and midwives 70.3%, 256/364).

#### Changes in Risk Assessment and Implications for Drug Therapy

A total of 66.6% (9694/14,562) of all users stated that they had changed their risk perception of a particular drug during pregnancy or breastfeeding after reading a fact sheet. Risk assessment remained unchanged more often in HCPs than in patients (physicians: 46.9%, 786/1676; pharmacists: 42.9%, 236/550; midwives: 51.9%, 189/364; and patients: 29.9%, 3252/10,860). Compared to their perception before reading the fact sheet, 16.2% (2358/14,562) of all users estimated the risk to be higher and 50.4% (7336/14,562) to be lower. The change of risk perception was also linked to the safety profile of the drug: After reading fact sheets with a red label on Embryotox, that is, fact sheets for drugs with known teratogenicity and/or severe fetotoxicity, 43.1% (81/188) of users estimated the risk to be higher (no change in risk assessment: 31.4%, 59/188). After reading fact sheets with a green label, that is, for drugs of choice for specified treatment indications, 58% (3642/6279) rated the risk to be lower (no change in risk assessment: 33%, 2070/6279). As a result of fact sheet information, 22.3% of users to whom the question applied (2252/10,083) considered changing drug treatment (“definitely yes”: 8.5%, 862/10,083; “possibly yes”: 13.8%, 1390/10,083). For physicians, the proportion was 27.9% (355/1274; with “definitely yes” 11.4%, 145/1274 and “possibly yes” 16.5%, 210/1274).

### Reasons to Trust and to Use Embryotox (Qualitative Interviews)

#### Overview

A total of 41 users were interviewed from February to November 2022, including 14 physicians and nine pharmacists, midwives, and patients each. Physicians worked in various specialized areas (gynecology, pediatrics, psychiatry, dentistry, and general medicine) and settings (hospitals and outpatient care). Ten physicians were female and 4 male, with a mean age of 41.8 (range 26-69) years. Most of the pharmacists worked in public pharmacies (8/9). Six were female and 3 were male, with a mean age of 37.8 (range 28-56) years. The majority of midwives worked freelance (8/9). All were female, with a mean age of 44.2 (range 34-56) years. Of the patients, 6 were pregnant, 2 were breastfeeding, and 1 patient was planning a pregnancy. Mean age was 34.6 (range 24-42) years.

#### Trust

All 41 interviewees stated to trust the information on Embryotox drug fact sheets. Interviewees from all user groups considered the association of Embryotox with a trustworthy institution to be a reason for trust, with the Charité – Universitätsmedizin Berlin and the Federal Ministry of Health mentioned most frequently. Users explicitly stated that they placed trust in the scientific basis of the website and perceived that drug fact sheets were developed by experts in the field. A physician summarized:

*It says ‘Federal Ministry of Health,’ ‘Charité university medicine’ … I mean, we are also a university hospital. So I think, university hospitals always work on a scientific basis and in this respect, these are all factors that constitute a certain independence and a certain scientific orientation, and that is what leads to trust*.

[Senior physician in obstetrics and gynecology, female, 41 years]

And a pharmacist stated:

... *that the website is somehow a well-known site and Charité Berlin is on it, that’s usually something you can rely on … they know how to read a study and how to interpret it*.

[Employee at a community pharmacy, female, 31 years]

In addition, the differentiated description of complex issues was named as a reason for trust, as a patient explained:

... *that the texts don't say you should definitely take a great amount or not at all, but that they try to provide a differentiated picture* ... .

[Patient, breastfeeding, 33 years]

Interviewees from all user groups trusted the Embryotox Center as the content provider of the website, which they explained by citing positive experiences with other Embryotox activities such as telephone counseling, lectures, and research projects. A midwife explained:

*So I think the phone contacts I’ve had so far, through these phone contacts, because there always... So, they went over it again, discussed it, explained it and that gives me a good feeling that I can trust the whole thing*.

[Freelancer, female, 38 years]

Furthermore, the absence of commercial interests and the long-standing experience of the Embryotox Center were given as reasons. Patients and pharmacists also emphasized that the recommendation of the website by other users, especially by physicians, built trust in the presented information. One patient said:

*So my midwife recommended it and my doctor recommended it and then, yes, I would trust the site ..., everything that is written there*.

[Patient, pregnant, 33 years]

Physicians said to trust the website because they had corroborated the information elsewhere, and also because of its clear structure and layout. One of them stated:

*The website also comes across as very serious, I would say. It’s not just some colorful [unintelligible] bouncing through the picture or something, but it’s ..., it strives to be attractive, but also serious*.

Further details on users’ assessment of trust can be found in Textbox 1.

Textbox 1. **Why do users trust the information on Embryotox? Reasons reported by users (based on 41 interviews, including 14 with physicians and 9 each with pharmacists, midwives, and patients).**

**Association with trustworthy institutions (interviewees of all user groups)**

- Embryotox Center, as the operator of the website, is part of Charité University Hospital (n*=*26)
- Funding by the German Ministry of Health (n*=*7)
- Perception of Embryotox as an “official” website (n*=*3)
- Website certified or funded by trustworthy organizations (n*=*2), including the “Health On the Net Foundation certificate (HONCode)’”, which was still available until December 2022, and the support from one of the major statutory health insurers in Germany at that time.

**Trust in Embryotox Center as operator of the website (interviewees of all user groups)**

- Drug fact sheets and recommendations developed by experts after detailed research (n*=*13)
- Long-standing experience and established position in the professional community (n*=*5)
- Positive experiences with telephone counseling on drug safety by the Embryotox Center (n*=*5)
- No commercial interests (n*=*7)
- Research projects conducted by Embryotox Center (n*=*4)
- No other similarly useful sources of information known (n*=*3)
- Participation in lectures by Embryotox experts (n*=*1)
- Absence of negative reports about Embryotox Center (n*=*1)

**Recommendation by other users (pharmacists and patients)**

- Use and recommendation by physicians (n*=*5)
- Use and recommendation by others, including nonphysician health care professionals (n*=*7)

**Scientific basis (physicians, pharmacists, and patients)**

- Drug fact sheets based on current scientific literature (n*=*14)

**Website content and layout (physicians and patients)**

- Content corroborated by clinical practice and own research (n*=*3)
- Differentiated description of complex issues (n*=*3)
- Clear structure and layout (n*=*2)
- Photos of staff on website (n=1)

#### Typical Use and Functions of Embryotox

Functions and purpose of typical fact sheet use considering different stakeholder groups are summarized in Textbox 2.

Textbox 2. **Typical use and functions of Embryotox for health care professionals and patients.**

**Health care professionals (based on interviews with 9 midwives, 9 pharmacists, and 14 physicians)**

- Researching information about drug treatment (13 physicians, 4 midwives, and 4 pharmacists)
- Corroborating information about drug treatment received earlier or elsewhere (8 physicians, 5 midwives, and 7 pharmacists)
- Informing, educating, and reassuring patients (9 physicians, 9 midwives, and 6 pharmacists)
- Pursuing an individual interest or professional development *(*4 physicians, 1 midwife, and 1 pharmacist)
- Documenting, communicating, or discussing patients’ drug treatment with colleagues on the basis of fact sheets (6 physicians)
- Challenging information a patient has received from a physician or pharmacist, or enabling the patient to challenge it (7 midwives)

**Patients (based on interviews with 9 patients)**

- Researching information about drug treatment (6 patients)
- Corroborating information about drug treatment received earlier or elsewhere (9 patients)
- Backup for discussing drug treatment with the attending physician or other people (4 patients)
- To reassure oneself (2 patients)

### Fact Sheet Use for Decision-Making (Questionnaire 2)

#### Overview

Of 1093 questionnaires, 793 were completed in full and 300 ended after 2 or 3 questions, because the decision-making process was still ongoing (214 patients) or fact sheets had not been used in the process of decision-making (23 physicians and 63 patients). The fully completed questionnaires (n=793) provided the data basis for further analyses; contributing user groups are shown in Table 3.

**Table 3.** Contributing users with fully completed questionnaire 2.

Completed questionnaires (N=793) by user group	Value, n (%)
Physicians	382 (48.2)
Medical specialties:	
Obstetrics and gynecology	96 (25.1)
General practitioner	78 (20.4)
Psychiatry, psychotherapy, or psychosomatics	67 (17.5)
Internal medicine	36 (9.4)
Pediatrics	25 (6.5)
Neurology	11 (2.9)
Other medical specialties	69 (18.1)
Patients	267 (33.7)
Pharmacists	79 (10)
Midwives	65 (8.2)

#### Typical Fact Sheet Use by HCP

To characterize their typical use of fact sheets, HCP could select a maximum of 3 out of 7 possible answers: 56.3% (215/382) of physicians used fact sheets typically before or after patient consultations, 76.3% (309/382) exclusively or additionally during the consultation. A total of 30.1% (93/309) of the latter routinely used fact sheets online together with their patients. A total of 46.9% (179/382) of physicians encouraged patients to inform themselves on Embryotox (gynecologists: 59.4%, 57/96) and 9.2% (35/382) printed out fact sheets for them (psychiatrists: 20.9%, 14/67). For further details, refer to Table S5 in Multimedia Appendix 1. Pharmacists and midwives used fact sheets to pass on selected information to patients (79.7%, 63/79 and 66.2%, 43/65, respectively), read the fact sheets together with patients (20.3%, 16/79 and 30.8%, 20/65), and encouraged patients to inform themselves on Embryotox (24.1%, 19/79 and 50.8%, 33/65). Fact sheets were also used to check the medication prescribed by physicians (pharmacists: 57%, 45/79; midwives: 52.3%, 34/65) and to consult with the prescribing physician if a medication was considered problematic (pharmacists: 58.2%, 46/79; midwives: 36.9%, 24/65).

**Figure 4.**
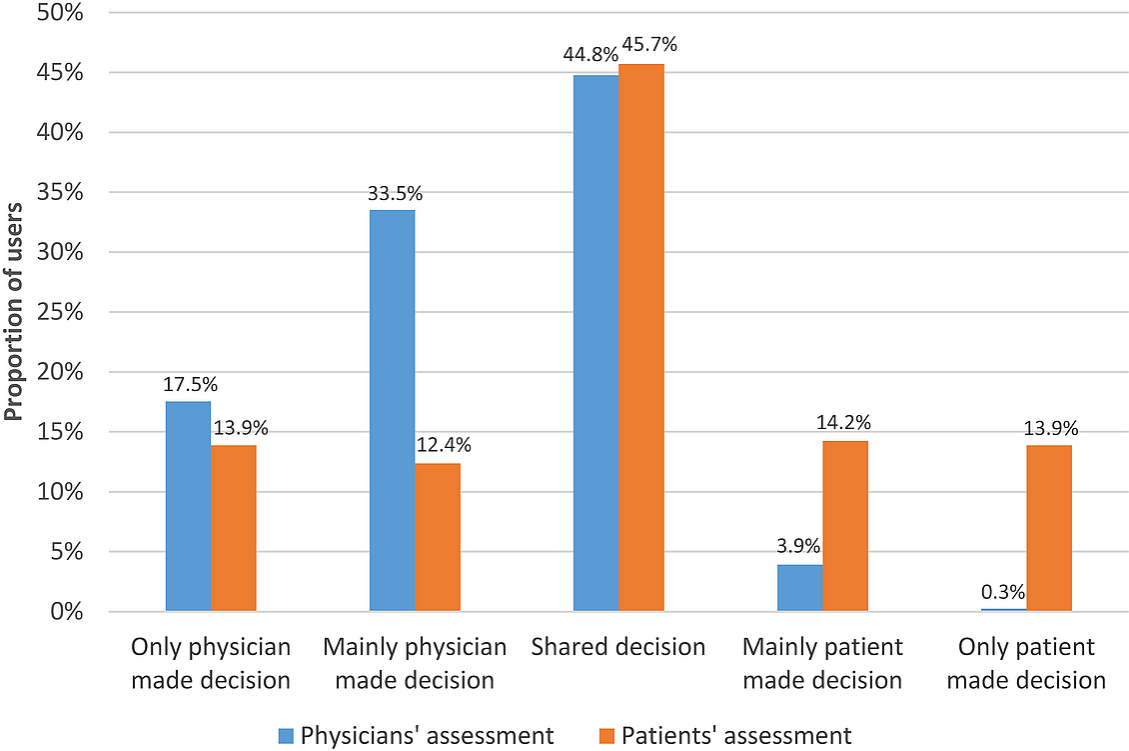
Physicians’ and patients’ assessment of who made the decision on drug therapy after using a fact sheet.

Figure 4 shows the extent to which physicians and patients considered themselves involved in making the final decision about drug therapy during pregnancy and breastfeeding after fact sheets have been used to obtain information for the decision-making process.

#### Benefits of Fact Sheet Use

**Figure 5.**
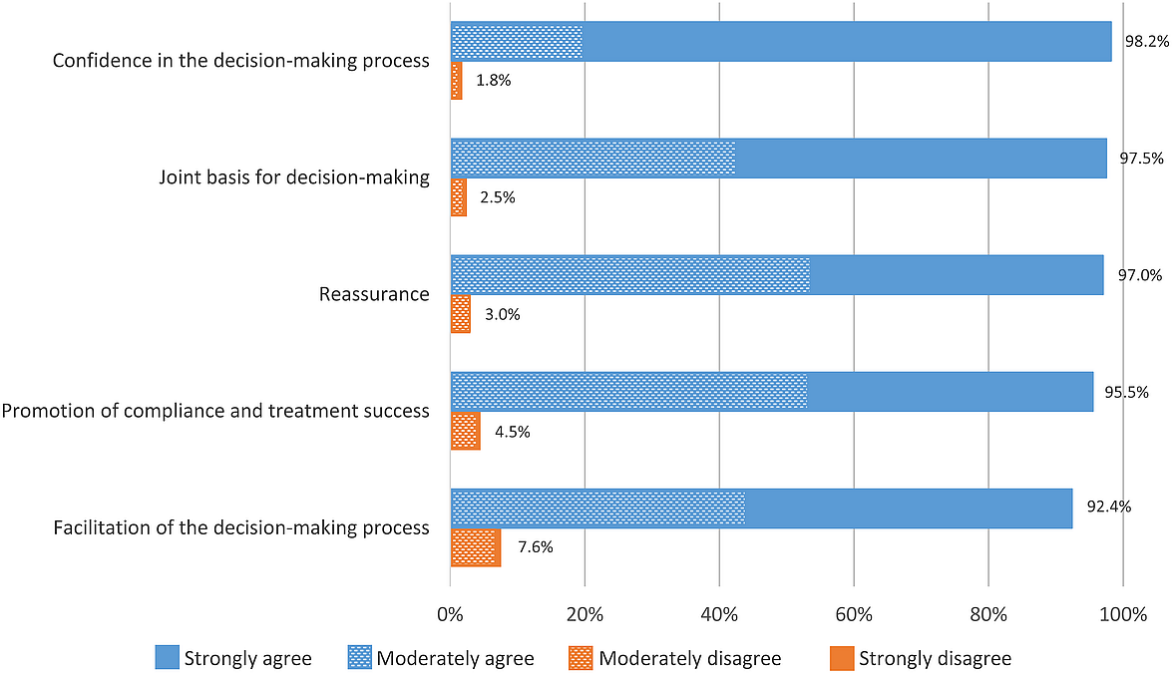
Benefits of the Embryotox fact sheet use for physicians. Percentages refer to all physicians except those who answered "question not applicable", that is, n=382 for all items except “Joint basis for decision making” (n=365) and “Reassurance” (n=369).

The majority of physicians and patients agreed to benefit from fact sheet use. Details are given in Figures5  6.

Patients also indicated that Embryotox drug fact sheets had helped them to convince their physician that a specific medication was compatible with pregnancy or breastfeeding (52.1%, 139/267) or that alternatives had to be considered (33%, 88/267). A total of 98.6% of pharmacists (71/72) agreed “moderately” or “strongly” to use Embryotox drug fact sheets for clarification in cases of disagreement between patients and physicians regarding drug therapy (question not applicable; n.a.: 7/79); the proportion for midwives was similar (95.2%, 59/62; n.a.: 3/65). Both user groups also indicated to use Embryotox drug fact sheets for informing and, if necessary, reassuring worried patients (pharmacists: 98.7%, 74/75; n.a.: 4/79; midwives: 100%, 62/62; n.a.: 3/65).

**Figure 6.**
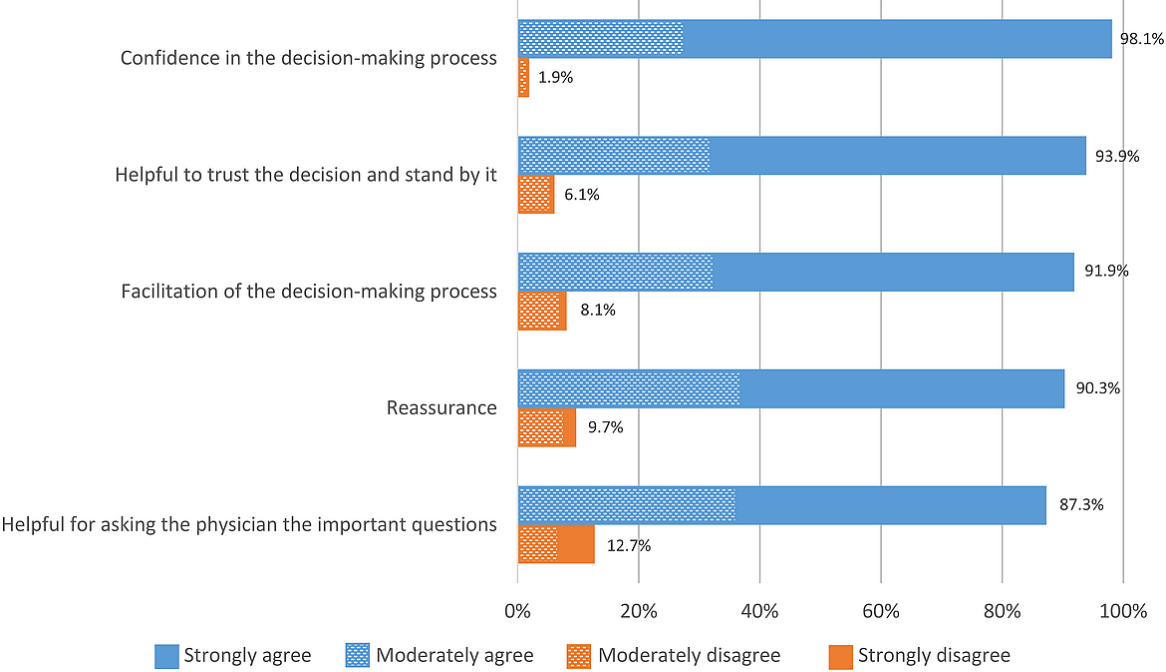
Benefits of the Embryotox fact sheet use for patients. Percentages refer to all patients except those who answered "question not applicable", that is, n=267 for all items except “Facilitation of the decision-making process” (n=236) and “Helpful to trust the decision and stand by it” (n=214).

#### Physicians’ Suggestions for Additional Online Content

A total of 53.4% (204/382) of physicians wished for more drug fact sheets on the website (gynecologists: 77.1%, 74/96). Furthermore, 42.9% (164/382) of physicians, particularly internists (55.6%, 20/36) and psychiatrists (47.8%, 32/67), considered “decision aids” to be useful, that is, structured guides through the decision-making process. Internet links to other reliable sources were requested by 19.6% (75/382), references to scientific literature by 14.1% (54/382), increased use of visualizations by 6.5% (25/382), and further explanations on given content by 4.7% (18/382). A total of 16.8% (64/382) did not consider any additions to be necessary.

## Discussion

### Principal Findings

For pregnant and breastfeeding women, as well as for their HCP, guidance on drug safety is among the most important health-related information needs [14,15,31-33]. To address this demand, publicly accessible, curated online knowledge resources on this topic have been established in several countries (see also Table S1 in Multimedia Appendix 1). To our knowledge, no comprehensive study has yet been conducted to establish how and by whom these databases are used, and whether they meet existing needs. The French resource [34] was evaluated in terms of appearance, content, interactivity, ease of use, and technical performance by 750 selected HCPs (including physicians, pharmacists, and midwives), who reported high levels of satisfaction [35,36]. However, the implementation of this and other publicly available resources in routine health care, and their use by patients and/or in shared decision-making has not yet been assessed. Thus, around 2 decades after the first online resources on drug safety during pregnancy and breastfeeding became available, our mixed methods study is the first to provide an in-depth, user group–specific evaluation of such a resource in routine health care.

The German website Embryotox was considered an established online resource for information on drug safety during pregnancy and breastfeeding. It was widely used 24/7 and viewed as trustworthy by HCPs and patients. This trust was largely based on the authority of the website operator and its affiliations—namely, the Embryotox Center as a specialist department of a university hospital—as well as on the scientific basis of the information. Both of these aspects align with the findings of existing research into how users seek and evaluate online health information [37-39]. Also consistent with previous studies [39,40], patients in our evaluation indicated that recommendations from their physicians and other HCPs to use the Embryotox website helped to build trust in the information. Given that trust is considered a key prerequisite for the patients’ intention to act on advice [41], the high level of trust in the information provided by Embryotox is vital to the website’s preventive impact.

The main user groups of the website were identified as patients, physicians, pharmacists, and midwives, accounting for 74.6%, 11.5%, 3.8%, and 2.5% of the 14,562 completed questionnaire 1 (Table 1). More than 70% (8937/12,536) of participating physicians and patients were repeat visitors to the fact sheets they accessed, suggesting a targeted search for updates and/or the need for reassurance. A relevant number of participating physicians searched for information before patients became pregnant, possibly to check whether a planned long-term medication would be compatible with pregnancy. This is particularly important given that around a third of all pregnancies in Germany are unplanned [42]. The high proportion of general practitioners as well as obstetricians and gynecologists among participating physicians (22.7% and 27.2%, respectively, in questionnaire 1) suggests that Embryotox is widely used in the primary health care of pregnant and breastfeeding women, thus promoting state-of-the-art drug treatment for common health issues. The fact sheets that are most frequently accessed by physicians—those relating to nonopioid analgesics and anti-infectives (Figure 1)—point in the same direction.

After reading a fact sheet, medications classified by Embryotox as drugs of choice for certain situations were perceived as less harmful, indicating a reassuring and adherence-promoting effect. Drugs classified as teratogenic and/or fetotoxic were perceived as more risky after reading the fact sheet, suggesting that the information was correctly understood as a safety warning. Both effects contribute to the safe use of medication during pregnancy and breastfeeding. Overall, around two-thirds of participating users changed their risk perception of a drug, and a significant proportion also considered changing drug treatment. These findings demonstrate the impact of the information provided by Embryotox in routine health care, as well as users’ intention to act based on the given information when necessary. Additionally, more than half of the participating physicians and over three-quarters of obstetricians and gynecologists would find further drug fact sheets useful, indicating the need for a broader, expanded range of information.

Comprehensibility of fact sheets was rated as good to very good, depending on user group and level of education: HCPs gave average ratings above 9 on a Likert scale from 0 to 10, while lay persons gave ratings above 8. However, patients with a higher level of education gave higher comprehensibility ratings and were overrepresented among users. These findings are in line with the results of other studies on the use of pregnancy-related internet resources [3] and telephone counseling services on drug safety in pregnancy [43]. Although Embryotox was initially developed for HCP, the high proportion of patients among users is not surprising, given that (1) the vast majority of pregnant women now search for health information online themselves; (2) there is no alternative, adequate German online resource; and (3) a large proportion of participating patients were referred to Embryotox by HCP—almost 40% (4283/10,860) by their physicians, others by their midwives or pharmacists (Figure 3). Despite there being no separate versions tailored specifically to the needs of each user group [44,45], our results show that both HCP and laypeople can effectively use Embryotox, albeit with the aforementioned limitations. In this context, it is important to note that Embryotox focuses on supporting very specific clinical decisions, which has already been discussed as a factor that facilitates the use of knowledge resources [46,47]. The fact that HCPs recommend the use of Embryotox to their patients could already indicate a potential role in shared decision-making. A 2023 umbrella review showed that involving patients in decision-making processes during pregnancy improved satisfaction with perinatal care, reduced distress, and strengthened the sense of empowerment and control [48]. Shared decision-making requires a collaborative process between physicians and patients, in which evidence-based medical information is discussed in the context of the patient’s individual values and priorities. Previous studies have shown that physicians would find it helpful to use online health information suitable for patient education [47], and that such information would support patients in making clinical decisions [49]. Therefore, it was not unexpected that patients in our study reported that using Embryotox empowered them to play a more active role in decision-making and sometimes even to alert their HCP to important options or information. Similarly, it was not unexpected that the joint use of Embryotox by all parties involved in the treatment process was reported to be of benefit to both HCPs and patients, as it facilitated communication and shared decision-making, strengthening confidence in the decision-making process (refer to Figures5  6 and Textbox 2). In this context, facilitating communication applies both to communication between HCPs and patients and to communication among HCPs, for example, between physicians of different medical areas or between physicians and midwives or pharmacists.

### Limitations

Overall, a solid database was available for both the descriptive statistics and the qualitative content analysis. However, quantitative data were collected in a convenience sample of website users who volunteered to participate. Therefore, the proportion of patients may be higher among participants than among all website users, as patients may be more interested in participating in online surveys than HCPs who are likely to be busier. In order to avoid bias in overall results due to a potentially disproportionate number of patients, results are generally reported by user group. It can be assumed that our study includes all relevant user groups, as residual categories such as “other HCP” or “other context” were rarely selected in the questionnaire. If, for example, users with positive Embryotox experiences used it more regularly and were more likely to participate, this may have introduced a degree of sampling bias (response bias). This could apply to patients with a higher level of education, who are overrepresented in our sample. The extent to which the use of Embryotox is appropriate for all relevant stakeholders in the care of pregnant and breastfeeding patients cannot be reliably described from our data, as the questionnaires were placed directly on the internet portal and only website users participated in the study. However, the steadily increasing number of visitors to the website, with more than 5 million visits in 2024, suggests widespread use in routine health care.

### Conclusions

The findings of this mixed methods study suggest that online knowledge resources on drug safety during pregnancy and breastfeeding, such as the freely accessible Embryotox website, can benefit a wide range of stakeholders in routine health care. With regard to Embryotox, further improvements in care could be achieved by increasing the number of available drug fact sheets and by making the online information easier to understand for less educated users. In its existing setup, users appreciated the website content for providing comprehensible information, facilitating communication, supporting shared decision-making, and empowering patients to take a more active role in decision-making. In doing so, Embryotox promotes treatment adherence and drug safety for pregnant and breastfeeding women.

## Supplementary material

10.2196/81286 Multimedia Appendix 1 Overview of online resources on drug safety in pregnancy and breastfeeding, study instruments, drug fact sheets, and survey results.

10.2196/81286 Multimedia Appendix 2 Questionnaire 1.

10.2196/81286 Multimedia Appendix 3 Questionnaire 2.

10.2196/81286 Checklist 1 CHERRIES checklist.

10.2196/81286 Checklist 2 COREQ checklist.

## Abbreviations

CHERRIES: Checklist for Reporting Results of Internet E-Surveys
COREQ: Consolidated Criteria for Reporting Qualitative Research
HCP: health care professional
