# Supplementary material for: Use of the Online Portal “Embryotox” in Routine Health Care: Mixed Methods Study
Source: J Med Internet Res. 2026 Jun 25;28:e81286. doi: 10.2196/81286 (PMC13299022; doi:10.2196/81286)
Supplement: Multimedia Appendix 1 [file jmir-v28-e81286-s001.docx]

# Supplement 1: Tables S1 – S5

## **Table S1.** Freely accessible, evidence-based online information on drug safety during pregnancy and breastfeeding (examples).

| **Responsible organization** | **Language** | **Online resource** |
| --- | --- | --- |
| Centre de Référence sur les Agents Tératogènes | French | http://www.lecrat.fr |
| IMI ConcePTION consortium (project funded by a private public partnership) | English, Polish,  Italian | https://www.mums.eu |
| Janusmed (Hälso- och sjukvårdsförvaltningen, Region Stockholm) | Swedish | https://janusmed.se/fosterpaverkan |
| Moeders van Morgen, Netherlands Pharmacovigilance Centre Lareb | Dutch | https://www.lareb.nl/mvm-kennis |
| Organization of Teratology Information Specialists (OTIS) | English, Spanish | https://mothertobaby.org/fact-sheets  https://mothertobaby.org/es/hojas-informativas |
| Pharmakovigilanz- und Beratungszentrum für Embryonaltoxikologie | German | https://www.embryotox.de |
| Regionale legemiddelinformasjonssentre (RELIS) | Norwegian | https://tryggmammamedisin.no |
| UK Teratology Information Service (UKTIS) | English | https://www.medicinesinpregnancy.org |
|  |  |  |

## **Table S2.** Overview of topics covered in the interview guides.*

|  | **Physicians** | **Pharmacists** | **Midwives** | **Patients** |
| --- | --- | --- | --- | --- |
| ***Professional background and environment*** | x | x | x | - |
| ***Personal situation regarding pregnancy and breastfeeding*** | - | - | - | x |
| ***Clinical circumstances of embryotox.de usage*** | x | x | x | x |
| ***Functions of embryotox.de use*** | x | x | x | x |
| ***Barriers of website use*** | x | x | x | x |
| ***Facilitators of website use*** | x | x | x | x |
| ***Website content, including comprehensibility*** | x | x | x | x |
| ***Trust in website content*** | x | x | x | x |
| ***Website structure and layout*** | x | x | x | x |
| ***Positive and negative experiences with using embryotox.de*** | x | x | x | x |
| ***Suggestions/wishes for optimization*** | x | x | x | x |
|  |  |  |  |  |

*The interview guides were adapted according to user group. In terms of clinical circumstances, for example, physicians were asked, among other things, about shared decision-making for prescription-only medication, and pharmacists were asked about counselling patients on the purchase of over-the-counter medication. The final project report containing the German interview guides will be published shortly (as of January 2026) by the third-party funder at https://innovationsfonds.g-ba.de/projekte/embryotox.390.

## **Table S3.** Factsheets on which questionnaire 2 was placed.*

| **Indication Category** | **Substances** |
| --- | --- |
| Anticoagulants | Argatroban, clopidogrel, danaparoid, fondaparinux, phenprocoumon |
| Antiepileptic drugs | carbamazepine, lamotrigine, levetiracetam, topiramate, valproate |
| Cardiovascular medications | amiodarone, amlodipine, atenolol, azilsartan, benazepril, bisoprolol, candesartan, captopril, carvedilol, digoxin, dihydralazine, enalapril, eprosartan, fosinopril, furosemid, hydrochlorothiazid, irbesartan, lisinopril, losartan, methyldopa, metildigoxin, metoprolol, moxonidine, nimodipine, nitrendipine, olmesartan, perindopril, propafenone, propranolol, quinapril, ramipril, telmisartan, urapidil, valsartan, verapamil |
| Immune modulating drugs | adalimumab, azathioprine, certolizumab pegol, ciclosporine, colchicine, cyclophosphamide, etanercept, dimethyl-fumarate, glatiramer, hydroxychloroquine infliximab, interferon beta-1a, interferon beta-1b, leflunomide, mercaptopurine, mesalazine, mycophenolate, omalizumab, prednisone, rituximab, sulfasalazine, vedolizumab |
| Prescription-only analgesics | buprenorphine, celecoxib, eletriptan, etoricoxib, fentanyl, frovatriptan, hydromorphone indomethacin, levo-methadone,  metamizole, methadone, morphine, oxycodone, pethidine, rizatriptan, tilidine and naloxone, tramadol |
| Psychotropic drugs | alprazolam, amisulpride, amitriptyline, aripiprazole, atomoxetine, benperidol, bromazepam, bupropion, buspirone, chlorprothixene, citalopram, clobazam, clomipramine, clonazepam, clostridium botulinum toxine, clozapine, diazepam, doxepin, duloxetine, escitalopram, flunitrazepam, fluoxetine, flupentixol, fluphenazine, fluspirilene, fluvoxamine, haloperidol, imipramine, l-dopa, levomepromazine, lithium, lorazepam, lormetazepam, maprotiline, melperon, methylphenidate, mianserin, midazolam, mirtazapine, moclobemide, nortriptyline, olanzapine, opipramol, paliperidone, paroxetine, perazine, perphenazine, pipamperone, pregabalin, promethazin, quetiapine, risperidone, sertraline, thioridazine, tranylcypromine, trimipramine, venlafaxine, ziprasidone, zolpidem, zopiclone, zuclopenthixol |
| Other | adapalene, atorvastatin, baclofen, benserazide, bromocriptine, cabergoline, carbidopa, carbimazole, clenbuterol, colestyramine, distigmine, domperidone, ebastine, ergotamine, fexofenadine, flunarizine, fluvastatin, glibenclamide, isotretinoin, lovastatin, metformin metoclopramide, mizolastine, neostigmine, ondansetron, oxybutynin, pentoxifylline, pravastatin, propylthiouracil, pyridostigmine, rupatadin, simvastatin, spironolactone, theophylline, thiamazole, tolperisone, tretinoin, ursodeoxycholic acid |

* Factsheets for drugs with systemic exposure which are only available on prescription and typically require shared decision-making.

## **Table S4.** Factsheet use by selected user groups (questionnaire 1).

|  | **Physicians** (n=1,676) | **Pharmacists** (n=550) | **Midwives**  (n=364) | **Patients**  (n=10,860) |
| --- | --- | --- | --- | --- |
| ***Day of the week when factsheets were used and questionnaire 1 completed*** | | | | |
| Working days (Monday to Friday) | 77.9% (n=1,306) | 80.7% (n=444) | 76.1% (n=277) | 75.1% (n=8,152) |
| Weekend (Saturday and Sunday) | 22.1% (n=370) | 19.3% (n=106) | 23.9% (n=87) | 24.9% (n=2,708) |
|  |  |  |  |  |
| ***Time of day when factsheets were used and questionnaire 1 completed*** | | | | |
| Standard working hours (8:00 a.m. to 7:59 p.m.) | 69.6% (n=1,167) | 71.6% (n=394) | 70.6% (n=257) | 57.1% (n=6,205) |
| Outside standard working hours (8:00 p.m. to 7:59 a.m.) | 30.4% (n=509) | 28.4% (n=156) | 29.4% (n=107) | 42.9% (n=4,655) |
|  |  |  |  |  |
| ***Number of visits to the evaluated drug factsheet on embryotox.de*** | | | | |
| First visit | 26.7% (n=447) | 30.9% (n=170) | 22.5% (n=82) | 29.0% (n=3,152) |
| Second or third visit | 15.9% (n=266) | 15.8% (n=87) | 15.1% (n=55) | 25.3% (n=2,745) |
| At least fourth visit | 57.5% (n=963) | 53.3% (n=293) | 62.4% (n=227) | 45.7% (n=4,963) |
|  |  |  |  |  |
| ***Research time on the evaluated drug factsheet*** | | | | |
| Less than 1 minute | 27.9% (n=468) | 26.9% (n=148) | 29.7% (n=108) | 21.9% (n=2,378) |
| 1 to 2 minutes | 38.0% (n=637) | 41.8% (n=230) | 36.0% (n=131) | 40.8% (n=4,428) |
| 2 to 5 minutes | 26.1% (n=438) | 24.0% (n=132) | 26.1% (n=95) | 27.9% (n=3,035) |
| More than 5 minutes | 7.9% (n=133) | 7.3% (n=40) | 8.2% (n=30) | 9.4% (n=1,019) |
|  |  |  |  |  |
| ***Research regarding a specific case*** | | | | |
| Yes | 82.8% (n=1,388) | 71.1% (n=391) | 86.3% (n=314) | n.a. |
| No (search for general information) | 17.2% (n=288) | 28.9% (n=159) | 13.7% (n=50) | n.a. |
|  |  |  |  |  |
| ***Clinical situation**** | | | | |
| Planning a pregnancy | 9.4% (n=131/1,388) | 5.1% (n=20/391) | 2.5% (n=8/314) | 6.0% (n=649/10,860) |
| Current pregnancy | 51.8% (n=719/1,388) | 53.5% (n=209/391) | 42.4% (n=133/314) | 46.8% (n=5,085/10,860) |
| Postpartum/newborn period | 4.0% (n=55/1,388) | 0.8% (n=3/391) | 3.5% (n=11/314) | 1.5% (n=166/10,860) |
| Breastfeeding | 30.2% (n=419/1,388) | 37.9% (n=148/391) | 48.7% (n=153/314) | 42.8% (n=4,650/10,860) |
| Developmental disorders or organ abnormalities in the child | 1.0% (n=14/1,388) | 0.3% (n=1/391) | 1.3% (n=4/314) | 0.9% (n=98/10,860) |
| Other | 3.6% (n=50/1,388) | 2.6% (n=10/391) | 1.6% (n=5/314) | 2.0% (n=212/10,860) |

* Data for all patients (n=10,860) and for healthcare professionals who researched on embryotox.de regarding a specific case.

## **T****able S5.** Physicians’ use of embryotox.de (questionnaire 2).

| **Typical use of embryotox.de (maximum 3 out of 6 answers possible)** | **All physicians**  **(n=382)** | **Gynecologists**  **(n=96)** | **Psychiatrists**  **(n=67)** | **Neurologists**  **(n=11)** | **General Practitioner**  **(n=78)** | **Internists**  **(n=36)** | **Pediatricians (n=25)** | **Other specialties (n=69)** |
| --- | --- | --- | --- | --- | --- | --- | --- | --- |
| Advising patients to inform themselves on embryotox.de, % (n) | 46.9%  (n=179) | 59.4%  (n=57) | 53.7%  (n=36) | 54.5%  (n=6) | 47.4%  (n=37) | 47.2%  (n=17) | 28.0%  (n=7) | 27.5%  (n=19) |
|  |  |  |  |  |  |  |  |  |
| Printing Embryotox drug factsheets and giving them to patients as information material, % (n) | 9.2% (n=35) | 9.4%  (n=9) | 20.9%  (n=14) | 18.2%  (n=2) | 6.4%  (n=5) | 0.0%  (n=0) | 12.0%  (n=3) | 2.9%  (n=2) |
|  |  |  |  |  |  |  |  |  |
| Use of embryotox.de before or after consultation/ward round, % (n) | 56.3%  (n=215) | 49.0%  (n=47) | 65.7%  (n=44) | 72.7%  (n=8) | 37.2%  (n=29) | 66.7%  (n=24) | 84.0%  (n=21) | 60.9%  (n=42) |
|  |  |  |  |  |  |  |  |  |
| Use of embryotox.de during consultation/ward round*, % (n) | 76.3%  (n=309) | 90.6%  (n=87) | 74.6%  (n=50) | 72.7%  (n=8) | 89.7%  (n=70) | 72.2%  (n=26) | 60.0%  (n=15) | 76.8%  (n=53) |

* Informing the patient of factsheet use for explaining the facts (also: looking at relevant drug factsheets on the screen with the patient) or without explicitly informing the patient.
