# Supplementary material for: Use of the Online Portal “Embryotox” in Routine Health Care: Mixed Methods Study
Source: J Med Internet Res. 2026 Jun 25;28:e81286. doi: 10.2196/81286 (PMC13299022; doi:10.2196/81286)
Supplement: Multimedia Appendix 2 [file jmir-v28-e81286-s002.docx]

# Questionnaire 1

#### Introductory text (all user groups)

**Please help us to improve embryotox.de.**

**Dear Embryotox user,**

**Please take 2-3 minutes to provide us with some feedback. Your responses will help us to improve embryotox.de.** **Please complete the questionnaire only once for each medicine.**

**Thank you for your support.**

**Your Embryotox team**

*[One answer option per question, unless otherwise indicated.]*

#### Today I am using embryotox.de as a… (all user groups)

- Physician *[1]*
- Patient *[2]*
- Pharmacist *[3]*
- Midwife *[4]*
- Other healthcare professional (e.g. nurse, medical or dental assistant) *[5]*
- Student of medicine or other health sciences *[6]*
- Partner/relative/friend of a patient *[7]*
- Another context *[8]*

#### Which age group do you belong to? (all user groups but [2])

- under 20
- 20-30
- 31-40
- 41-50
- 51-60
- 61-70
- over 70

#### Which age group do you belong to? (user group [2])

- under 20
- 20-30
- 31-40
- 41-50
- over 50

#### Please indicate your gender: (all user groups but [2])

- female
- male
- diverse

#### Where do you work? (user groups [1], [3], [4], [5])

- Hospital
- Outpatient care (physician’s office/other outpatient setting)
- Pharmacy
- Other area

#### What medical specialty do you work in? (user group [1])

- Gynecology and obstetrics
- Psychiatry/psychotherapy/psychosomatics
- Neurology
- Family doctor/general practitioner
- Internal medicine (possibly with further specialization)
- Pediatrics, including neonatology
- Human genetics
- Dentistry
- Other specialization/other area of expertise

#### How did you learn about the internet portal embryotox.de? (Multiple answers possible) (user groups [2], [7], [8])

- Own online research
- Recommendation by physician
- Recommendation by midwife
- Recommendation by pharmacist
- Mention in the media or on other websites
- Recommendation by friends, family or acquaintances
- Other

#### What is your highest educational qualification? (Optional) (user groups [2], [7], [8])

- No educational qualification (yet)
- School-leaving certificate (9th grade, “Hauptschulabschluss”)
- School-leaving certificate (10th/11th grade, “Mittlere Reife”)
- Qualification for higher education (“(Fach-)Abitur”)
- Higher education/academic degree

#### How many times have you visited the factsheet for this drug? (all user groups)

- Today for the first time
- Already 2 or 3 times
- More often

#### How long did you spend researching this drug today? (all user groups)

- Less than 1 minute
- 1 to 2 minutes
- More than 5 minutes

#### Which sections have you read? (Multiple answers possible) (all user groups)

- Evidence for use during pregnancy: 1st trimester
- Evidence for use during pregnancy: 2nd/3rd trimester
- Recommendations for pregnancy
- Breastfeeding
- If described: paternal use

#### Did you find the necessary information about this drug to make a well-founded treatment decision? (user groups [1], [3], [4], [5], [6])

No, not at all. – Not really. – Somewhat. – Yes, definitely.

#### Was the information understandable for you? (all user groups)

not understandable at all

completely understandable

0 1 2 3 4 5 6 7 8 9 10

#### After reading the factsheet for this drug, and compared to what I knew before, I now estimate the risk of this drug during pregnancy and/or breastfeeding to be... (all user groups)

Significantly higher. – Slightly higher. – Slightly lower. – Significantly lower. – My risk assessment has not changed.

#### Are you looking for information regarding a specific case? (user groups [1], [3], [4], [5], [6], [8])

- Yes, I am researching with reference to a specific patient/child.
- No, I am searching for general information. *[🡪 Thank you for your feedback. The questionnaire is now completed.]*

#### If "Yes" in the previous question, questions a) and b) follow:

#### a) Today I am mainly researching the following situation:

- Planning a pregnancy
- Current pregnancy
- Postpartum/newborn period
- Breastfeeding
- Developmental disorders or congenital abnormalities in the child *[*🡪 *Thank you for your feedback. The questionnaire is now completed.]*
- Other

#### b) Is there already a concrete plan for drug treatment?

- Yes, drug treatment has already started.
- Yes, but drug treatment has not yet started.
- Drug treatment is currently being planned.
- No, so far there is no concrete plan.
- I don't know.

#### Today I am mainly researching the following situation (user groups [2], [7])

- Planning a pregnancy
- Current pregnancy
- Postpartum/newborn period
- Breastfeeding
- Developmental disorders or organ abnormalities in the child *[🡪 Thank you for your feedback. The questionnaire is now completed.]*
- Other

#### Were specific drugs suggested to the patient by her physician? (user group [7])

- Yes, drug treatment has already started.
- Yes, but drug treatment has not yet started.
- Drug treatment is currently being planned.
- No, so far there is no concrete plan.
- I don't know.

#### Have any specific drugs been suggested to you by your physician? (user group [2])

- Yes, drug treatment has already started.
- Yes, but drug treatment has not yet started.
- Drug treatment is currently being planned.
- No, so far there is no concrete plan.

Do you think the treatment should be changed based on the information in this drug factsheet? *(*user groups *[2] and [7]; if questions a) and b) were answered: [1], [3], [4], [5], [6] und [8])*

Definitely not. – Probably not. – Possibly yes. – Definitely yes. – Question not applicable.

**Thank you for your feedback. The questionnaire is now completed.**
