# Supplementary material for: Use of the Online Portal “Embryotox” in Routine Health Care: Mixed Methods Study
Source: J Med Internet Res. 2026 Jun 25;28:e81286. doi: 10.2196/81286 (PMC13299022; doi:10.2196/81286)
Supplement: Multimedia Appendix 3 [file jmir-v28-e81286-s003.docx]

# Questionnaire 2

#### Introductory text (all user groups)

**Dear user,**

**With the following questions, we aim to find out how Embryotox drug factsheets are utilized for decision-making about the use of prescription-only medicines during pregnancy planning, pregnancy and breastfeeding.**

**Please only take part in the survey if you belong to one of the following user groups:**

#### Today I am using embryotox.de as a… (all user groups)

- Physician
- Patient
- Pharmacist
- Midwife

## Questions for physicians

#### Which medical specialty do you work in?

- Gynecology and obstetrics
- Psychiatry/psychotherapy/psychosomatics
- Neurology
- Family doctor/general practitioner
- Internal medicine (possibly with further specialization)
- Pediatrics, including neonatology
- Other specialization/other area of expertise

#### How do you usually use Embryotox drug factsheets? (Please select a maximum of 3 options)

- I advise the patient to inform herself on embryotox.de.
- I print out Embryotox drug factsheets and hand them to patients as information material.
- I inform myself through embryotox.de before or after the consultation/ward round.
- I use embryotox.de as a source of information for myself during the consultation/ward round, without explicitly discussing this with the patient.
- I inform the patient that I am using embryotox.de and explain the facts using the relevant drug factsheets.
- I inform the patient that I use embryotox.de and explain the facts while we take a look at the relevant drug factsheets on the screen together.
- Not at all. *[🡪 The questionnaire is now completed, thank you very much for your contribution!
  Your Embryotox team.]*

#### How do you usually assess the benefits and risks of a drug therapy?

0 1 2 3 4 5 6 7 8 9 10

I assess benefits and risks together with the patient.

I assess benefits and risks by myself as a physician.

#### Who makes the decision about drug therapy during pregnancy and breastfeeding after Embryotox

#### drug factsheets have been used to obtain information for the decision-making process?

Physician – Mainly physician – Physician and patient – Mainly patient – Patient

#### Are you generally satisfied with the decision-making process using embryotox.de?

- Yes.
- No, I would appreciate more patient involvement.
- No, I would prefer patients to be less involved in decisions about drug therapy.

#### Please evaluate the following statements based on your experience:

#### When making a risk-benefit assessment before starting a drug therapy, the Embryotox drug factsheets provide a joint basis for decision-making.

Strongly agree. – Moderately agree. – Moderately disagree. – Strongly disagree. – Question
not applicable.

#### Decision-making requires more time if the patient informs herself using Embryotox drug factsheets.

Strongly agree. – Moderately agree. – Moderately disagree. – Strongly disagree.

#### The use of Embryotox drug factsheets by the physician and patient together facilitates the decision-making process.

Strongly agree. – Moderately agree. – Moderately disagree. – Strongly disagree.

#### Embryotox drug factsheets provide confidence in the decision-making process.

Strongly agree. – Moderately agree. – Moderately disagree. – Strongly disagree.

#### The information on Embryotox drug factsheets makes patients feel insecure.

Strongly agree. – Moderately agree. – Moderately disagree. – Strongly disagree.

#### Embryotox drug factsheets enhance patient compliance/adherence and thus contribute to treatment success.

Strongly agree. – Moderately agree. – Moderately disagree. – Strongly disagree.

#### By using Embryotox drug factsheets, worried patients can be reassured.

Strongly agree. – Moderately agree. – Moderately disagree. – Strongly disagree. – Question not applicable.

#### There are no drug factsheets on embryotox.de for the drugs I need information about.

Strongly agree. – Moderately agree. – Moderately disagree. – Strongly disagree.

#### Please select one or two options that you would consider useful in addition to the information provided by embryotox.de:

- In my opinion, no additions are required.
- New drug factsheets for further drugs.
- Structured guidelines for substance-specific benefit-risk assessments (“decision aids”).
- Information about literature references.
- More explanations on given content.
- Links to further reliable information sources on the Internet.
- Increased use of visualizations.

**The questionnaire is now completed, thank you very much for your contribution!**

**Your Embryotox team.**

## Questions for patients

#### Introductory text (for patients)

**The questions refer to the drug therapy you have just researched.**

#### How did you use embryotox.de?

- I used embryotox.de proactively or on the advice of non-physicians.
- My physician advised me to look up information on embryotox.de.
- My physician did research on embryotox.de and then informed me.
- My physician and I searched for information on embryotox.de together during the consultation/ward round visit.
- Not at all. *🡪 The questionnaire is now completed, thank you very much for your contribution!
  Your Embryotox team.*

#### How were benefits and risks of the drug assessed?

0 1 2 3 4 5 6 7 8 9 10

The benefits and risks of drug therapy were discussed in detail in consultation with my physician.

I assessed the benefits and risks of drug therapy without my physician.

#### Has a decision on drug therapy during pregnancy planning, pregnancy or breastfeeding already been made?

- No. *🡪 The questionnaire is now completed, thank you very much for your contribution!
  Your Embryotox team.*
- Yes.

#### Who made the decision?

Physician – Mainly physician – Physician and I – Mainly me – Me

#### Were you satisfied with the decision-making process?

- Yes.
- No, I would have liked to be more involved in the decision-making process.
- No, I would have liked the physician to be more involved.

#### Please evaluate the following statements:

#### The Embryotox drug factsheets helped me to ask my physician questions that were important to me.

Strongly agree. – Moderately agree. – Moderately disagree. – Strongly disagree.

#### The use of the Embryotox drug factsheet facilitated the decision-making process with the physician regarding drug therapy.

Strongly agree. – Moderately agree. – Moderately disagree. – Strongly disagree. – Question not applicable.

#### The Embryotox drug factsheets give me confidence in the decision-making process.

Strongly agree. – Moderately agree. – Moderately disagree. – Strongly disagree.

#### Using the Embryotox drug factsheets, I convinced my physician that prescribing a specific medication is compatible with pregnancy/breastfeeding.

Strongly agree. – Moderately agree. – Moderately disagree. – Strongly disagree.

#### Using the Embryotox drug factsheets, I convinced my physician that a specific medication is not compatible with pregnancy/breastfeeding and alternatives need to be found.

Strongly agree. – Moderately agree. – Moderately disagree. – Strongly disagree.

#### The information on the Embryotox drug factsheets has made me feel insecure.

Strongly agree. – Moderately agree. – Moderately disagree. – Strongly disagree.

#### If you have decided to take a medication based on the recommendation of your physician: Do the Embryotox drug factsheets help you to trust and stand by the decision?

Strongly agree. – Moderately agree. – Moderately disagree. – Strongly disagree. – Question not applicable.

#### The information on the Embryotox drug factsheets reassured me.

Strongly agree. – Moderately agree. – Moderately disagree. – Strongly disagree.

#### There are no drug factsheets on embryotox.de for the drugs I need information about.

Strongly agree. – Moderately agree. – Moderately disagree. – Strongly disagree.

#### Did you discuss the decision with anyone else? (Multiple answers possible)

- No.
- Yes, with my partner.
- Yes, with a maximum of three relatives or friends.
- Yes, with more than three relatives or friends.
- Yes, with my midwife.
- Yes, with a pharmacist.
- Yes, with other physicians.

**The questionnaire is now completed, thank you very much for your contribution!**

**Your Embryotox team.**

## Questions for pharmacists

#### In which situation do you typically use Embryotox drug factsheets to advise patients?

- Pregnancy planning.
- Pregnancy.
- Breastfeeding.

#### For which medication do you typically use Embryotox drug factsheets?

- Mainly for non-prescription medication.
- Mainly to check whether medication prescribed by a physician may be used during pregnancy or breastfeeding.
- Equally for both medication prescribed by a physician and non-prescription medication.

#### How do you usually use the Embryotox drug factsheets when counseling pregnant or breastfeeding patients? (Please select a maximum of 3 options)

- I look up the drug factsheets and pass on specific information to the patient.
- I obtain information from the drug factsheets together with the patient.
- I refer the patient to the drug factsheets so that she can inform herself.
- I use the drug factsheets to consult with the prescribing physician in case of problematic drugs.
- I use the drug factsheets for myself to check the medication prescribed by a physician before I give the medication to the patient.

#### Please evaluate the following statements:

#### If there are disagreements between patients and physicians regarding drug therapy during pregnancy/breastfeeding, I try to seek clarification using the Embryotox drug factsheets.

Strongly agree. – Moderately agree. – Moderately disagree. – Strongly disagree. – Question not applicable.

#### Embryotox factsheets help me to inform worried patients and reassure them if necessary.

Strongly agree. – Moderately agree. – Moderately disagree. – Strongly disagree. – Question not applicable.

**The questionnaire is now completed, thank you very much for your contribution!**

**Your Embryotox team.**

## Questions for midwives

#### In which situation do you typically use Embryotox drug factsheets to advise patients?

- Pregnancy planning.
- Pregnancy.
- Breastfeeding.

#### For which medication do you typically use Embryotox drug factsheets?

- Mainly for non-prescription medication.
- Mainly to check whether medication prescribed by a physician may be used during pregnancy or breastfeeding.
- Equally for both medication prescribed by a physician and non-prescription medication.

#### How do you usually use the Embryotox drug factsheets when counseling pregnant or breastfeeding patients? (Multiple answers possible if different options are used depending on need)

- I look up the drug factsheets and pass on specific information to the patient.
- I obtain information from the drug factsheets together with the patient.
- I refer the patient to the drug factsheets so that she can inform herself.
- I use the drug factsheets to consult with the prescribing physician in case of problematic drugs.
- I use the drug factsheets for myself to check the medication prescribed by a physician.

#### Please evaluate the following statements:

#### If there are disagreements between patients and physicians regarding drug therapy during pregnancy/breastfeeding, I try to seek clarification using the Embryotox drug factsheets.

Strongly agree. – Moderately agree. – Moderately disagree. – Strongly disagree. – Question not applicable.

#### Embryotox factsheets help me to inform worried patients and reassure them if necessary.

Strongly agree. – Moderately agree. – Moderately disagree. – Strongly disagree. – Question not applicable.

**The questionnaire is now completed, thank you very much for your contribution!**

**Your Embryotox team.**
