# Supplementary material for: Use of the Online Portal “Embryotox” in Routine Health Care: Mixed Methods Study
Source: J Med Internet Res. 2026 Jun 25;28:e81286. doi: 10.2196/81286 (PMC13299022; doi:10.2196/81286)
Supplement: Checklist 1 [file jmir-v28-e81286-s004.docx]

## Checklist for Reporting Results of Internet E-Surveys (CHERRIES)^[[1]](#footnote-1)^

| ***Checklist Item*** | ***Explanation*** | ***Details for the present study*** |
| --- | --- | --- |
| Describe survey design | Describe target population, sample frame. Is the sample a convenience sample? (In “open” surveys this is most likely.) | Target population: users of the website embryotox.de.  Sample: convenience sample. |
| IRB approval | Mention whether the study has been approved by an IRB. | Ethical approval for collection and analysis of qualitative and quantitative data were obtained from the Ethical Committee of the Charité – Universitätsmedizin Berlin (EA1/334/21). |
| Informed consent | Describe the informed consent process. Where were the participants told the length of time of the survey, which data were stored and where and for how long, who the investigator was, and the purpose of the study? | Participants were informed about the time needed to complete the questionnaires, the investigator and the purpose of the study. The online questionnaires did not request personal data that would allow for identification of the participants. To participate, users actively had to open the questionnaire, to select their answers and to select the button “Send data” after completion, otherwise answers were not saved. The risk to participants was negligible, and formal written consent was not required, as confirmed by the Ethics Committee of Charité – Universitätsmedizin Berlin (EA1/334/21). |
| Data protection | If any personal information was collected or stored, describe what mechanisms were used to protect unauthorized access. | The online questionnaires did not request personal data that would allow for identification of the participants. |
| Development and testing | State how the survey was developed, including whether the usability and technical functionality of the electronic questionnaire had been tested before fielding the questionnaire. | The two online questionnaires were developed by the project team and tested among colleagues from both academic and non-academic backgrounds, as well as among individuals from their social circles. Technical details were worked out in collaboration with the digital agency that technically manages the website and also programmed both questionnaires. |
| Open survey versus closed survey | An “open survey” is a survey open for each visitor of a site, while a closed survey is only open to a sample which the investigator knows (password-protected survey). | Open survey. |
| Contact mode | Indicate whether or not the initial contact with the potential participants was made on the Internet. (Investigators may also send out questionnaires by mail and allow for Web-based data entry.) | Initial contact was made on the internet. |
| Advertising the survey | How/where was the survey announced or advertised? Some examples are offline media (newspapers), or online (mailing lists – If yes, which ones?) or banner ads (Where were these banner ads posted and what did they look like?). It is important to know the wording of the announcement as it will heavily influence who chooses to participate. Ideally the survey announcement should be published as an appendix. | Banner ads leading to the questionnaires were based directly on the drug factsheets on the website. The banner leading to the first questionnaire (2022) invited users to participate with the words “We ask for your feedback.”. On the banner leading to the second questionnaire (2023) users were asked, “How do you use embryotox.de? Questionnaire about your experiences.”. |
| Web/E-mail | State the type of e-survey (eg, one posted on a Web site, or one sent out through e-mail). If it is an e-mail survey, were the responses entered manually into a database, or was there an automatic method for capturing responses? | The questionnaires could be accessed directly from the website. |
| Context | Describe the Web site (for mailing list/newsgroup) in which the survey was posted. What is the Web site about, who is visiting it, what are visitors normally looking for? Discuss to what degree the content of the Web site could pre-select the sample or influence the results. For example, a survey about vaccination on a anti-immunization Web site will have different results from a Web survey conducted on a government Web site | The publicly accessible online platform embryotox.de provides evidence-based information on drug safety during pregnancy and lactation. The evaluation of user characteristics was one of the objectives of the study. |
| Mandatory/voluntary | Was it a mandatory survey to be filled in by every visitor who wanted to enter the Web site, or was it a voluntary survey? | Voluntary survey. |
| Incentives | Were any incentives offered (eg, monetary, prizes, or non-monetary incentives such as an offer to provide the survey results)? | No. |
| Time/Date | In what timeframe were the data collected? | 3 May 2022 until 13 December 2022. During this period, data exports for interim evaluations were carried out approximately once a month. |
| Randomization of items or questionnaires | To prevent biases items can be randomized or alternated. | None of these measures were taken. |
| Adaptive questioning | Use adaptive questioning (certain items, or only conditionally displayed based on responses to other items) to reduce number and complexity of the questions. | Adaptive questioning was used, meaning certain items or answers were displayed based on previous answers. |
| Number of Items | What was the number of questionnaire items per page? The number of items is an important factor for the completion rate. | The number of questions varied depending on previous answers. There was only one question at a time displayed on the screen, and only when a question had been answered, participants could move on to the next question (exception: optional question on educational background). |
| Number of screens (pages) | Over how many pages was the questionnaire distributed? The number of items is an important factor for the completion rate. | The number of screens varied depending on previous answers; detailed flowcharts are found in Supplements 2 and 3. |
| Completeness check | It is technically possible to do consistency or completeness checks before the questionnaire is submitted. Was this done, and if “yes”, how (usually JAVAScript)? An alternative is to check for completeness after the questionnaire has been submitted (and highlight mandatory items). If this has been done, it should be reported. All items should provide a non-response option such as “not applicable” or “rather not say”, and selection of one response option should be enforced. | The selection of mutually exclusive answer options and the selection of more than one answer (if not explicitly requested) was blocked. The answers were only saved if the users completed the questionnaire to the end and then selected the button “Send data”. Technical details were worked out in collaboration with the digital agency that technically manages the website and also programmed both questionnaires. All questions except the question on educational background were mandatory, most complex questions contained answer options like “question not applicable”, “others” etc. |
| Review step | State whether respondents were able to review and change their answers (eg, through a Back button or a Review step which displays a summary of the responses and asks the respondents if they are correct). | Respondents were not able to change their answers once they had progressed, but all answers were only saved if the user completed the questionnaire to the end and then selected the button “Send data”. |
| Unique site visitor | If you provide view rates or participation rates, you need to define how you determined a unique visitor. There are different techniques available, based on IP addresses or cookies or both. | Visits on *Embryotox* drug factsheets were counted via an anonymous session tracking by a commercial provider. Overall, a median of 0.58% (IQR 0.42 – 0.77) of visits to a drug factsheet resulted in the completion of questionnaire 1. |
| View rate (Ratio of unique survey visitors/unique site visitors) | Requires counting unique visitors to the first page of the survey, divided by the number of unique site visitors (not page views!). It is not unusual to have view rates of less than 0.1 % if the survey is voluntary. | The questionnaires could be accessed from all drug factsheets (questionnaire 1) or specific drug fact sheets (questionnaire 2) on the online portal. However, we cannot provide information on survey non-completers to give an exact view rate. |
| Participation rate (Ratio of unique visitors who agreed to participate/unique first survey page visitors) | Count the unique number of people who filled in the first survey page (or agreed to participate, for example by checking a checkbox), divided by visitors who visit the first page of the survey (or the informed consents page, if present). This can also be called “recruitment” rate. | The questionnaires could be accessed from all drug factsheets (questionnaire 1) or specific drug fact sheets (questionnaire 2) on the online portal. However, we cannot provide information on survey non-completers to give an exact participation rate. |
| Completion rate (Ratio of users who finished the survey/users who agreed to participate) | The number of people submitting the last questionnaire page, divided by the number of people who agreed to participate (or submitted the first survey page). This is only relevant if there is a separate “informed consent” page or if the survey goes over several pages. This is a measure for attrition. Note that “completion” can involve leaving questionnaire items blank. This is not a measure for how completely questionnaires were filled in. (If you need a measure for this, use the word “completeness rate”.) | We do not have detailed information on survey non-completers. |
| Cookies used | Indicate whether cookies were used to assign a unique user identifier to each client computer. If so, mention the page on which the cookie was set and read, and how long the cookie was valid. Were duplicate entries avoided by preventing users access to the survey twice; or were duplicate database entries having the same user ID eliminated before analysis? In the latter case, which entries were kept for analysis (eg, the first entry or the most recent)? | To identify potential multiple entries, data sets of questionnaires were analyzed regarding entry times and similarity of entries. IP addresses or cookies were not used for this purpose. |
| IP check | Indicate whether the IP address of the client computer was used to identify potential duplicate entries from the same user. If so, mention the period of time for which no two entries from the same IP address were allowed (eg, 24 hours). Were duplicate entries avoided by preventing users with the same IP address access to the survey twice; or were duplicate database entries having the same IP address within a given period of time eliminated before analysis? If the latter, which entries were kept for analysis (eg, the first entry or the most recent)? | To identify potential multiple entries, data sets of questionnaires were analyzed regarding entry times and similarity of entries. IP addresses or cookies were not used for this purpose. |
| Log file analysis | Indicate whether other techniques to analyze the log file for identification of multiple entries were used. If so, please describe. | To identify potential multiple entries, data sets of questionnaires were analyzed regarding entry times and similarity of entries. |
| Registration | In “closed” (non-open) surveys, users need to login first and it is easier to prevent duplicate entries from the same user. Describe how this was done. For example, was the survey never displayed a second time once the user had filled it in, or was the username stored together with the survey results and later eliminated? If the latter, which entries were kept for analysis (eg, the first entry or the most recent)? | Not applicable. |
| Handling of incomplete questionnaires | Were only completed questionnaires analyzed? Were questionnaires which terminated early (where, for example, users did not go through all questionnaire pages) also analyzed? | Only completed questionnaires were saved and analyzed. |
| Questionnaires submitted with an atypical timestamp | Some investigators may measure the time people needed to fill in a questionnaire and exclude questionnaires that were submitted too soon. Specify the timeframe that was used as a cut-off point, and describe how this point was determined. | The time people needed to fill in a questionnaire was not measured. |
| Statistical correction | Indicate whether any methods such as weighting of items or propensity scores have been used to adjust for the non-representative sample; if so, please describe the methods. | Only descriptive statistics were used. |

1. Eysenbach G. Improving the quality of Web surveys: the Checklist for Reporting Results of Internet E-Surveys (CHERRIES). J Med Internet Res. 2004 Sep 29;6(3):e34. PMID: 15471760. doi: 10.2196/jmir.6.3.e34; erratum available at <https://www.jmir.org/2012/1/e8/>. [↑](#footnote-ref-1)
