# Supplementary material for: Use of the Online Portal “Embryotox” in Routine Health Care: Mixed Methods Study
Source: J Med Internet Res. 2026 Jun 25;28:e81286. doi: 10.2196/81286 (PMC13299022; doi:10.2196/81286)
Supplement: Checklist 2 [file jmir-v28-e81286-s005.docx]

## COREQ Checklist

|  | **Details for the present study** |
| --- | --- |
| **Domain 1: Research team and reflexivity** |  |
| *Personal Characteristics* |  |
| 1. Interviewer/facilitator,   Which author/s conducted the interview or focus group? | A.M. |
| 1. Credentials,   What were the researcher´s credentials? (e.g. PhD, MD) | Dr. rer. nat. (interviewer), for the research team see affiliations. |
| 1. Occupation,   What was their occupation at the time of the study? | Research fellow (interviewer), other qualitative researchers: physicians (K.D., F.M., M.O., C.S.), psychologist (M.B.), professorship in social medicine and epidemiology (C.H.). |
| 1. Gender,   Was the researcher male or female? | Female (interviewer), qualitative research team: female and male. |
| 1. Experience and training,   What experience or training did the researcher have? | A.M. has experiences as a researcher and interviewer in previous qualitative studies. Among the other qualitative researchers, there were experts with extensive scientific and publishing experience in qualitative and mixed-methods methodology (MB and CH). |
| *Relationship with participants* |  |
| 1. Relationship established,   Was a relationship established prior to study commencement? | No. |
| 1. Participant knowledge of the interviewer,   What did the participants know about the researcher? (e.g. personal goals, reasons for doing the research) | Participants were not informed about personal motivation of the interviewer. |
| 1. Interviewer characteristics,   What characteristics were reported about the interviewer/facilitator? (e.g. bias, assumptions, reasons and interests in the research topic) | The interviewer introduced herself as research fellow not involved in the daily counselling work of the *Embryotox Center*. |
| **Domain 2: Study design** |  |
| *Theoretical framework* |  |
| 1. Methodological orientation and Theory,   What methodological orientation was stated to underpin the study? (e.g. grounded theory, discourse analysis, ethnography, phenomenology, content analysis) | Thematic qualitative content analysis with a deductive-inductive approach was employed to analyze the material as described by Kuckartz (Kuckartz U. Qualitative Text Analysis: A Guide to Methods, Practice & Using Software. London: SAGE Publications Ltd; 2014). |
| *Participant selection* |  |
| 1. Sampling,   How were participants selected? (e.g. purposive, convenience, consecutive, snowball) | Interviewees were recruited by purposive and snowball sampling. The aim of the sampling was to achieve broad representation of the interviewees’ experiences. |
| 1. Method of approach,   How were participants approached? (e.g. face-to-face, telephone, mail, email) | By phone. |
| 1. Sample size,   How many participants were in the study? | 41 participants. |
| 1. Non-participation,   How many people refused to participate or dropped out? Reasons? | No one dropped out after the interview had been conducted. Those who did not want to participate were not assessed. |
| *Setting* |  |
| 1. Setting of data collection,   Where was the data collected? (e.g. home, clinic, workplace) | During the phone interview, interviewer and interviewees were in a separate room at home or at their workplace. |
| 1. Presence of non-participants,   Was anyone else present besides the participants and researchers? | No. |
| 1. Description of sample,   What are the important characteristics of the sample? (e.g. demographic data, date) | All 41 interviewees were users of embryotox.de. 14 were physicians, nine were pharmacists, nine were midwives, and nine were patients. |
| *Data collection* |  |
| 1. Interview guide,   Were questions, prompts, guides provided by the authors? Was it pilot tested? | Interview guides were developed by the authors. Following an iterative process involving pilot interviews, adjustments were made for the relevant user groups and agreed by consensus within the project team. |
| 1. Repeat interviews,   Were repeat interviews carried out? If yes, how many? | No. |
| 1. Audio/visual recording,   Did the research use audio or visual recording to collect the data? | Audio recording. |
| 1. Field notes,   Were field notes made during and/or after the interview or focus group? | No. |
| 1. Duration,   What was the duration of the interviews or focus group? | The interviews lasted approximately 30 minutes. |
| 1. Data saturation,   Was data saturation discussed? | The occurrence of new themes was assessed with each new interview within the respective user group, allowing us to evaluate saturation of topics. The sampling and interviewing process for the respective user group was concluded, if few or no new topics had emerged in the last three interviews. |
| 1. Transcripts returned,   Were transcripts returned to participants for comment and/or correction? | No. |
| **Domain 3: Analysis and findings** |  |
| *Data analysis* |  |
| 1. Number of data coders,   How many data coders coded the data? | The initial coding was done by two researchers, disagreements were solved by discussion within the project team. |
| 1. Description of the coding tree,   Did authors provide a description of the coding tree? | The main topical categories were developed deductively from the interview guide. Categories and subcategories were determined inductively from the material. The coding tree and themes were double-checked and discussed in the project team to guarantee inter-subjective comprehensibility |
| 1. Derivation of themes,   Were themes identified in advance or derived from the data? | The main topical categories were developed deductively from the interview guide. Categories and subcategories were determined inductively from the material.­­­ |
| 1. Software,   What software, if applicable, was used to manage the data? | f4transkript/analyse software (f4transkript v7 and f4analyse v3, dr. dresing & pehl GmbH, Marburg, Germany) |
| 1. Participant checking,   Did participants provide feedback on the findings? | No. |
| *Reporting* |  |
| 1. Quotations presented,   Were participant quotations presented to illustrate the themes/findings? Was each quotation identified? (e.g. participant number) | Quotations were presented to illustrate the findings; for each quotation the user group to which the interviewee belonged was specified. |
| 1. Data and findings consistent,   Was there consistency between the data presented and the findings? | Yes. |
| 1. Clarity of major themes,   Were major themes clearly presented in the findings? | Yes, two major themes were presented in the findings and subsequently discussed (user trust and functions of factsheet use). |
| 1. Clarity of minor themes,   Is there a description of diverse cases or discussion of minor themes? | Due to the scope of this manuscript, presenting the approach and overview results, two major topics were selected. Any other major or minor topics will be published in a separate manuscript. |
